# Pediatric Collagenous Gastroduodenitis: A Rare Cause of Iron-Deficiency Anemia

**DOI:** 10.7759/cureus.72939

**Published:** 2024-11-03

**Authors:** Palack Agrawal, Keshav Bhattar, Claudia Rojas, Jacqueline Larson

**Affiliations:** 1 Pediatrics, Joe DiMaggio Children's Hospital, Hollywood, USA; 2 Internal Medicine, Memorial Healthcare, Hollywood, USA; 3 Anatomic and Clinical Pathology, Pediatric Pathology, Memorial Healthcare, Hollywood, USA; 4 Pediatric Gastroenterology, University of South Florida Morsani College of Medicine, Tampa, USA

**Keywords:** anemia, collagenous duodenitis, collagenous gastritis, collagenous gastroenteritides, jejunum

## Abstract

Collagenous gastroenteritidesare rare disorders of unknown etiology diagnosed histologically by marked subepithelial deposition of collagen bands thicker than 10µm in the lamina propria with a mononuclear inflammatory infiltrate. Collagenous gastritis (CG) is divided into two phenotypes - pediatric-onset and adult-onset. Up until recently, pediatric-onset CG was thought to be confined to the stomach presenting with abdominal pain and anemia with limited involvement of the colon. Whereas adult-onset CG is often associated with involvement of the small and/or large intestine presenting with chronic non-bloody diarrhea and weight loss. It is now acknowledged that adult-onset and pediatric-onset CG should be considered a similar disease on a continuous spectrum. There are limited case reports of pediatric patients diagnosed as CG with concurrent collagenous duodenitis (CD) and/or collagenous colitis (CC). There are no accepted therapeutic standards for treating these patients. We present a rare case of an adolescent male with selective IgA deficiency and growth hormone deficiency presenting with severe iron deficiency anemia and abdominal pain with an ultimate diagnosis of collagenous gastroduodenitis with suspected jejunal involvement.

## Introduction

Collagenous gastroenteritides are rare disorders of unknown etiology diagnosed histologically by marked subepithelial deposition of collagen bands thicker than 10µm in the lamina propria with a mononuclear inflammatory infiltrate [1]. Collagenous colitis (CC) is well supported in the literature in the adult population. The proximal collagenous gastroenteritides - collagenous gastritis (CG) and collagenous sprue/duodenitis (CD) - are less recognized and likely overlooked. There have been reports that collagen deposition may be a response to an inflammatory, autoimmune, infectious, medication, or toxic insult [2-5]. Due to the rarity and/or underreporting of these conditions, there is limited understanding of the etiology, pathophysiology, and treatment options.

Collagenous duodenitis (CD) was first described in 1947 and formally introduced in 1970, CC in 1979, and CG in 1989 [6-8]. Collagenous gastritis (CG) is divided into two phenotypes, pediatric-onset and adult-onset [2,5]. Up until recently, pediatric-onset CG was thought to be confined to the stomach presenting with abdominal pain and anemia with limited involvement of the colon [5,8]. Whereas adult-onset CG is often associated with involvement of the small and/or large intestine presenting with chronic non-bloody diarrhea and weight loss [5,8]. It is now acknowledged that adult-onset and pediatric-onset CG should be considered a similar disease on a continuous spectrum [2]. There are limited pediatric case reports of patients diagnosed as CG with concurrent CD and/or CC [3,9-13].

There are no accepted therapeutic standards for treating adult or pediatric patients. Depending on location of disease, different treatment strategies with variable outcomes have included topical targeted budesonide (TTB), corticosteroids, H2-receptor antagonists, proton pump inhibitors, dietary allergen restriction, oral and IV iron therapy, sucralfate, bismuth subsalicylate, mesalamine, sulfasalazine, azathioprine, and anti-tumor necrosis factor (TNF) inhibitors [2,3,5,8-10,14-16].

We present a rare case of a pediatric patient with selective IgA deficiency without a history of chronic infections, growth hormone deficiency, and strep throat months prior to presentation with severe iron deficiency anemia and abdominal pain with an ultimate diagnosis of collagenous gastroduodenitis with suspected jejunal involvement.

## Case presentation

A 15-yo male with selective IgA deficiency without chronic infections and growth hormone deficiency managed with growth hormone (GH) for 6 years, presented for a second opinion for worsening anemia and abdominal pain with diffuse nodular gastritis confirmed on esophagogastroduodenoscopy (EGD) by an outside provider.

Two months prior to the original presentation to an outside provider, the patient was diagnosed with strep throat. Over the following two months, he started to experience abdominal pain, dizziness, pallor, and palpitations. Initial complete blood count revealed hemoglobin (Hb) 6.8 grams/deciliter (g/dl) (normal (n) 12-16.9g/dl), hematocrit (Hct) 21.5%, mean corpuscular volume (MCV) 65.8 femtoliters (fL), and platelets 282,000/microliter (mcL). The patient was directed to the emergency department and subsequently admitted. Hematology work-up found normal hemoglobin electrophoresis, lactate dehydrogenase, creatinine phosphokinase, uric acid, complete metabolic profile, sedimentation rate (ESR), and C-reactive protein (CRP). Iron studies were indicative of iron deficiency anemia with iron 18 micrograms/dl (mcg/dl) (n 27-164 mcg/dl), total iron binding capacity (TIBC) 572 mcg/dl (n 271-448 mcg/dl), iron saturation 3 (n 16-48), and ferritin undetectable at <4.5 nanograms/milliliter (ng/ml) (n 11-172 ng/ml). The patient received two units packed red blood cells and two IV sucrose infusions.

The patient underwent EGD with findings of erosive nodular gastritis of the antrum, body, and fundus, and nodularity of the duodenal bulb. Biopsies were limited to the antrum and the second part of the duodenum. Histology confirmed mild active gastritis, negative *Helicobacter pylori*, and nonspecific chronic duodenitis. The patient was discharged home on sucralfate and omeprazole.

The patient was re-evaluated at our center two months later for persistent epigastric pain and fatigue. Additional information was obtained; the patient was born in the United States and is of Eastern European ancestry. Autoimmunity in the family includes the mother with psoriatic arthritis and the maternal grandfather with psoriasis.

**Figure 1:**
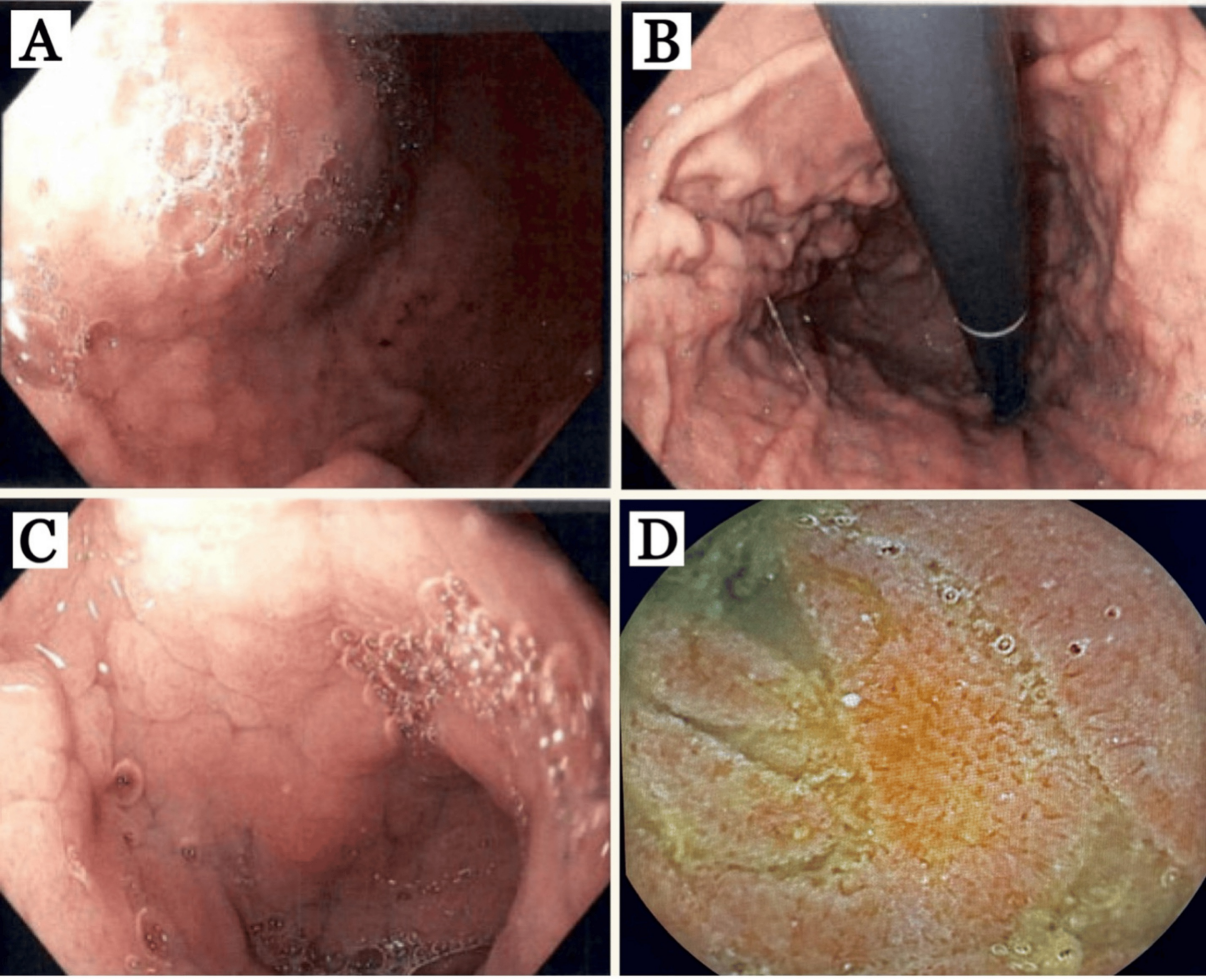
Esophagogastroduodenoscopy findings (A, B, C) and pill cam (D). (A) Gastric body and (B) fundus: diffuse nodularity, erythema, and swelling. (C) Duodenal bulb: diffuse nodularity, swelling, erythema, fissured appearance, villous blunting, and few aphthous ulcers. (D) Proximal jejunum: erythema, swelling, and villous blunting.

The patient underwent repeat EGD in addition to colonoscopy and pill cam. Upper endoscopy findings included gastric antrum, body (Figure 1A), and fundus (Figure 1B) with diffuse nodularity, swelling, erythema, and scattered superficial erosions, duodenal bulb (Figure 1C) with diffuse swelling, erythema, nodularity, fissured appearance, flattened villi, and few aphthous ulcerations, second and third portions of the duodenum were normal, pill cam of the proximal jejunum (Figure 1D) with diffuse erythema, swelling and villous blunting, entire colon and terminal ileum were all normal.

**Figure 2:**
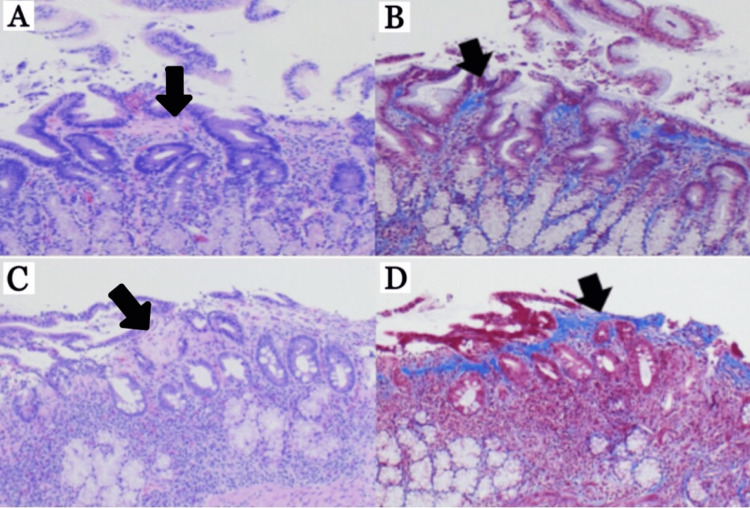
Histopathological examination. (A) Gastric antrum: 40 eosinophils/high-powered field (hpf) in the lamina propria and thickened subepithelial collagen layer (arrow), (B) gastric antrum: positive trichome stain for subepithelial collagen deposition (arrow), (C) duodenal bulb: 45 eosinophils/hpf in the lamina propria, thickened subepithelial collagen layer (arrow), and villous blunting (D) duodenal bulb: positive trichome stain for subepithelial collagen deposition (arrow).

Histopathology findings included gastric antrum (Figure 2A, 2B) with active chronic gastritis, 40 eosinophils per high-powered field (hpf) in the lamina propria (LP), thickened subepithelial collagen layer, and positive trichome stain for subepithelial collagen deposition, duodenal bulb (Figure 2C, 2D) with 45 eosinophils/hpf in the LP, thickened subepithelial collagen layer, villous blunting, and positive trichome stain for subepithelial collagen deposition. Not pictured: gastric fundus with 15 eosinophils/hpf in the LP and thickened subepithelial collagen layer, and gastric body with active chronic gastritis, 47 eosinophils/hpf in the LP, and thickened subepithelial collagen layer.

New laboratory studies (after five IV sucrose infusions) with Hb 12.4 g/dl, Hct 41.3%, iron 39 mcg/dl, TIBC 421 mcg/dl, iron saturation 9, and ferritin 29.80 ng/ml. Additional labs included negative human leukocyte antigen (HLA)DQ2 and DQ8, IgG celiac screen, given the patient’s selective IgA deficiency (normal tissue transglutaminase IgG, endomysial antibody IgG, gliadin IgG), normal ESR, CRP, thyroid stimulating hormone, free T4, and fecal calprotectin. 

**Figure 3:**
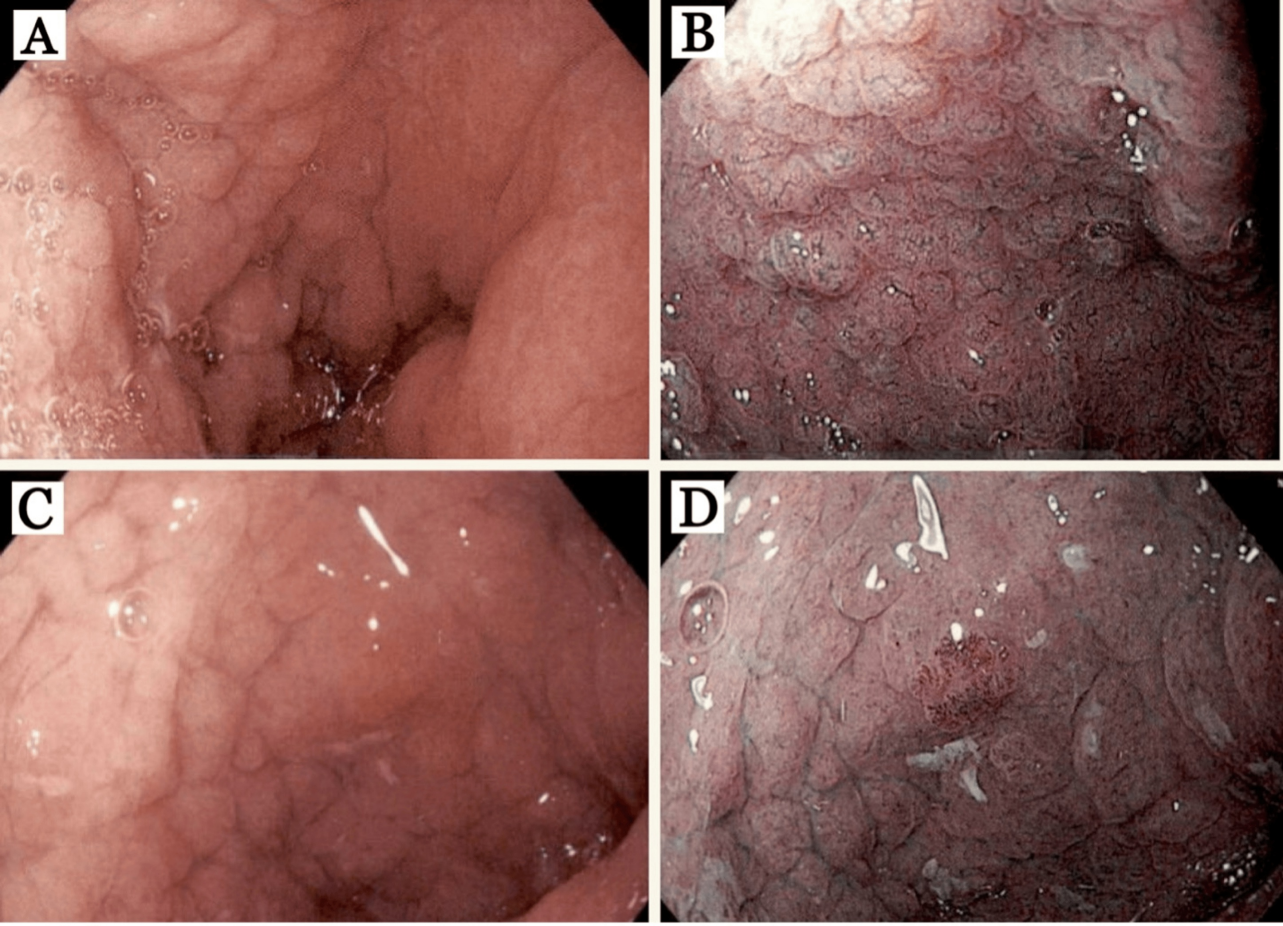
Esophagogastroduodenoscopy findings after 8 weeks of budesonide 9 mg. (A) Gastric body: diffuse nodularity, erythema, and swelling, (B) gastric body: narrow-band imaging (NBI) showing the mucosal surface of the nodular lesions without marked changes and abnormal capillary vessels. The depressed mucosa surrounding the nodules shows no changes and no abnormal capillary vessels. The depressed mucosa surrounding the nodular lesions shows an amorphous or absent surface structure and abnormal capillary vessels. (C) Duodenal bulb: diffuse nodularity, swelling, erythema, villous blunting, fissured appearance, and few aphthous, (D) Duodenal bulb: NBI showing the same findings as (B) gastric body.

**Figure 4:**
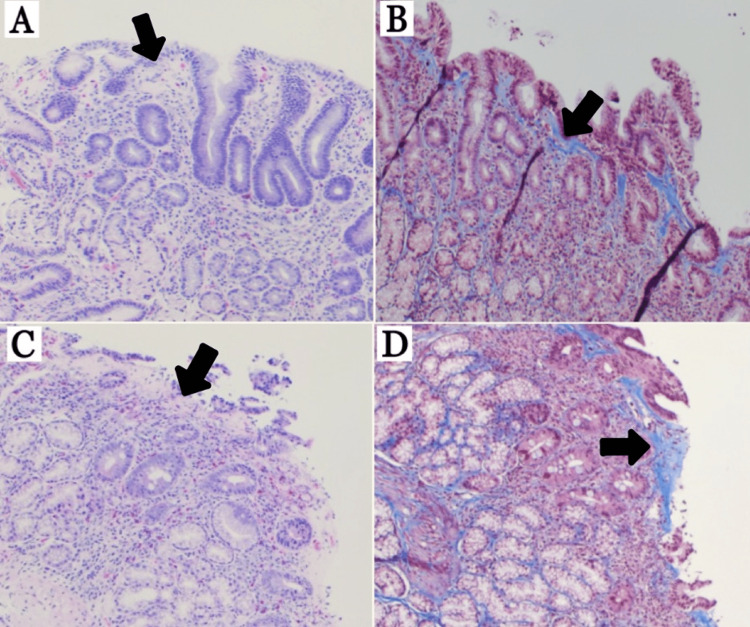
Histopathological examination after 8 weeks of budesonide 9 mg. (A) Gastric body: 30 eosinophils/hpf in the lamina propria, thickened subepithelial collagen layer (arrow), (B) gastric body: positive trichome stain for subepithelial collagen deposition (arrow), (C) duodenal bulb: 70 eosinophils/hpf in the lamina propria, villous blunting, and thickened subepithelial collagen layer (arrow), (D) duodenal bulb: positive trichome stain for subepithelial collagen layer (arrow).

The patient was placed on crushed budesonide EC 9 mg mixed with water once daily given at bedtime and omeprazole 40 mg twice daily. After 8 weeks, the patient had significant improvement in abdominal pain and anemia (Hb 13.2 g/dl, Hct 43.4%, iron 61 mcg/dl, TIBC 399 mcg/dl, iron saturation 15, ferritin 16.60 ng/ml). Repeat EGD (Figure 3) revealed mild endoscopic improvement in the gastric antrum, body (Figure 3A), and fundus with decreased nodularity and erythema. Narrow band imaging (NBI) (Figure 3B) of the gastric body showed the mucosal surface of the nodular lesions without marked changes or abnormal capillary vessels. The depressed mucosa surrounding the nodular lesions showed no changes and no abnormal capillary vessels. The depressed mucosa surrounding the nodular lesions showed an amorphous or absent surface structure and abnormal capillary vessels. The duodenal bulb (Figure 3C, 3D) remained unchanged and the 2nd and 3rd portions of the duodenum remained normal. Histopathology (Figure 4) was virtually unchanged - gastric body (Figure 4A,4 B) with 30 eosinophils/hpf in the LP, thickened subepithelial collagen layer, and positive trichome stain for subepithelial collagen deposition, duodenal bulb (Figure 4C, 4D) with 70 eosinophils/hpf in the LP, thickened subepithelial collagen layer, villous blunting, and positive trichome stain for subepithelial collagen deposition. 

In view of clinical improvement and patient’s request, budesonide EC was weaned to 6 mg for four weeks followed by 3 mg for four weeks. Labs after four weeks of 3 mg budesonide with Hb 11.4 g/dl, iron 34 mcg/dl, TIBC 440 mcg/dl, iron saturation 8, and ferritin 2 ng/ml. The patient ultimately stopped the budesonide and continued omeprazole 40 mg once daily. The patient’s anemia worsened and he was treated with two ferric carboxymaltose injections due to the development of intolerance to IV sucrose. The patient is currently undergoing an immunological work-up.

## Discussion

Proximal collagenous gastroenteritides are rare conditions diagnosed histologically by marked subepithelial deposition of collagen bands thicker than 10 µm in the lamina propria with a mononuclear inflammatory infiltrate. Due to the rarity of these conditions, the cause and pathogenesis are not well understood. Collagen deposition has been postulated to be an outcome of underlying injury and chronic inflammation, which could result from an underlying condition or insult [4,14,17].

Clinical symptoms are variable with the pediatric type presenting with abdominal pain and anemia as in this case, and the adult type presenting with watery diarrhea given concurrent involvement of the large intestine. Multiple case reports show an overlap of symptoms between the pediatric and adult types [2]. Proximal collagenous gastroenteritides should be considered in patients of any age presenting with anemia, epigastric pain, dyspepsia, weight loss, and/or diarrhea, particularly when upper endoscopy shows nodularity of the gastric and/or duodenal mucosa.

Collagenous gastroenteritides are diagnosed histologically and endoscopic appearance can mimic that of *Heliobacter pylori*. In this case, the first endoscopy took place with an outside provider and did not identify collagenous gastroduodenitis. Biopsies were limited to the antrum and the second part of the duodenum. There are no current recommendations on the number of biopsies to take or where to take the biopsy for the diagnosis of collagenous gastroenteritides. Our center follows similar approaches for the diagnosis of celiac disease and eosinophilic gastrointestinal disorders (EGIDs) given endoscopic appearance is often normal and histologic findings can be patchy. Eight biopsies are taken from the stomach (two-fundus, two-body, four-antrum), and six biopsies from the duodenum/bulb (four-duodenum, two-duodenal bulb). Narrow band imaging (NBI) studies have examined gastric mucosa in CG patients finding no changes in the mucosal surface of the nodular lesions. The changes have instead been found in the depressed mucosa surrounding the nodular lesions indicating that the depressed mucosal pattern is the result of inflammation with atrophic changes and collagen deposition, whereas the nodular lesions are the remaining undamaged mucosa [18]. Assuring proper sampling from multiple areas and a biopsy of the depressed mucosal pattern will maximize the diagnostic sensitivity, ensure a quick and accurate diagnosis, and provide the best patient outcomes.

Associations with immunodeficiency and autoimmune conditions have been found, particularly celiac disease [4,5,19]. This is relevant in this case as the patient has selective IgA deficiency. IgA-deficient patients are 10 to 20 times more likely to develop an autoimmune response to gluten when compared to the general population. There have been reports of CG and CD patients responding to a gluten-free diet [7,19,20]. A gluten-free diet was discussed with the patient.

Medications have been proposed as another potential cause for collagen deposition [2-5]. This patient has been on GH therapy for 6 years. Growth hormone is important for growth, tissue remodeling, extracellular matrix formation, and fibrosis [21]. Growth hormone has been investigated for wound healing and collagen synthesis in both human and animal studies. Doessing et al found GH caused a rise in matrix collagen synthesis of skeletal muscle and tendon [22]. Thorey et al found after an injury to the skin, the process of wound healing is accelerated in GH-transgenic mice overexpressing GH [23]. In this case, one hypothesis includes compromise to the gastric and duodenal mucosa from strep throat two months prior to presentation with resulting collagen deposition from the patient's growth hormone therapy [4,14,17]. Repeat EGD is being planned after the patient has been off GH therapy for at least 6 months.

There are no accepted therapeutic standards for treating these patients with treatment based on small patient series and case reports. Treatment is often directed towards symptom control and iron therapy for anemia. The degree of anemia in these patients can be significant given the location of iron absorption, duodenum, and proximal jejunum. For the treatment of CG, the most promising therapy to date is TTB [5,14]. Many patients have a resolution of symptoms and anemia with treatment. There have been mixed results in the endoscopic appearance on repeat endoscopy. Some patients show persistent endoscopic abnormalities while other patients show remission or resolution of clinical symptoms over time [5,14]. Histopathologic reversal has been more difficult to substantiate owing to the focal, sometimes extensive nature, of this pathologic process [4].

## Conclusions

Proximal collagenous gastroenteritides are rare disorders with a variable clinical presentation depending on the patient’s age and the location of the disease. Patients can present with abdominal pain, nausea, vomiting, chronic diarrhea, weight loss, and anemia. These symptoms can overlap with other disorders such as celiac disease, inflammatory bowel disease, and eosinophilic gastrointestinal disorders. The degree of anemia in these patients can be profound given the location of iron absorption in the duodenum and proximal jejunum. Profound anemia with or without abdominal pain and without obvious rectal bleeding should raise suspicion for collagenous gastroenteritides. 

As more cases are reported that do not fit the typical pediatric and adult phenotypes, evaluation with EGD, colonoscopy, and pill cam should be considered. There are no accepted therapeutic standards to treat these patients and the current strategy is to control symptoms and treat the anemia. This case highlights the importance of accurately taking biopsies of both normal and abnormal areas, repeating endoscopy when symptoms persist, and regular follow-up. In order, to advance the understanding of these rare conditions, monitoring of disease with repeat endoscopy in the asymptomatic patient should be considered.
